# Event-related brain potentials that distinguish false memory for events that occurred only seconds in the past

**DOI:** 10.1186/1744-9081-8-36

**Published:** 2012-07-30

**Authors:** Hong Chen, Joel L Voss, Chunyan Guo

**Affiliations:** 1 Beijing Key Laboratory of Learning and Cognition, Department of Psychology, Capital Normal University, Beijing 100048, China; 2 Center of College Students’ Mental Health Guidance, Xi’an University of Architecture and Technology, Xi’an 710055, China; 3 Department of Medical Social Sciences, Northwestern University Feinberg School of Medicine, Chicago, IL, USA

**Keywords:** DRM, False memory, Short-term memory, Priming, ERP

## Abstract

**Background:**

False memory often involves retrieving events from the distant past that did not actually happen. However, recent evidence obtained using the Deese/Roediger-McDermott (DRM) paradigm for eliciting false memory experiences suggests that individuals can falsely believe that events occurred mere seconds in the past when they in fact did not. Subjects in these experiments endorsed unstudied critical lure words as having been studied, despite the fact that word lists were studied just moments before. We identified event-related brain potential (ERP) correlates of this experience, and included a repetition priming manipulation to better assess the functional significance of these ERPs.

**Methods:**

Behavioral and ERP data were collected from 21 Capital Normal University students using a short-term DRM task.

**Results:**

Two categories of effects were identified that distinguished true from false short-term memory: (1) early semantic priming effects from 300 to 500 ms and (2) later retrieval and retrieval-monitoring effects after 500 ms. The repetition priming manipulation had distinct influences on these effects, consistent with their differential associations with semantic priming versus episodic retrieval.

**Conclusion:**

Characterization of ERPs related to semantic priming and episodic retrieval provides important information regarding the mechanisms of short-term false memory. In contrast, most studies examining false memory in standard long-delay DRM paradigms identify ERP effects related only to retrieval monitoring. These findings highlight the neural processing involved in illusions of memory after very brief delays and highlight the role of semantic processing in short-term false memory.

## Background

Subjectively compelling memory experiences can occur even for events that never happened, a phenomenon referred to as false memory [1]. Many laboratory studies of false memory have used the Deese-Roediger-McDermott (DRM) paradigm [2,3]. The DRM paradigm involves presenting a thematically organized list of words (associates), each semantically related to a non-presented critical item (a lure). During subsequent memory testing, subjects often falsely report that the non-presented critical lure was studied earlier, often with high confidence. As with most false memory experiences, DRM false memory events generally occur after an appreciable delay from the events that are (falsely) remembered, as DRM paradigms often involve study-test delays of at least several minutes and with intervening study sessions before the critical tests. This experimental approach is consistent with real-world false memory experiences, in which false memory tends to involve relatively distant events, such as from childhood [4]. However, it is also possible to induce false memory using the DRM paradigm when the memory test follows the study experience by only several seconds and with no intervening study experience [5-8]. Remarkably, subjects demonstrate robust false-memory effects at these brief study-test intervals, thus begging the question of what kind of cognitive and neural processing can cause individuals to falsely claim that just seconds before they read words that were not actually presented to them.

Some evidence for the processes related to false memory in standard, long-delay DRM experiments has come from event-related brain potential (ERP) studies. For instance, positive ERPs maximal at parietal electrodes from about 400–800 ms after stimulus onset are more positive for true memory (endorsements of studied items) than for false memory (endorsement of unstudied items) [9-11]. These parietal potentials, known generally as the late-positive complex (LPC) are related to the retrieval of episodic details from study [12], and could indicate that retrieval of these details is greater for studied items than for false memory events. Indeed, in the studies noted above, LPC amplitudes were no greater for falsely endorsed critical lures than for correct rejections of novel items unrelated semantically to the study lists, suggesting that LPC retrieval effects are not indicative of false memory. In contrast, ERPs with an onset after 800 ms have been shown to distinguish true memory, false memory, and correct endorsement of novel items, with the greatest amplitude for true memory and intermediate amplitude for false memory [11,13-18]. These late potentials at frontal and parietal electrodes are thought to reflect post-retrieval evaluation processing, thus implicating a general monitoring process that is more effective for true memory than for false memory.

In the current experiment, we examined ERP correlates of true and false memory using a short-term version of the DRM paradigm. Our goal was to identify neurocognitive processing relevant for false memory at this interval. Based on the recognition memory ERP literature, we made several predictions about the nature of neural correlates of short-term false memory and how these neural correlates could suggest both similar and dissimilar neurocognitive processing relative to false memory in long-delay DRM paradigms (see also [19]). For example, only a small number of long-delay DRM experiments have identified an N400-like ERP component associated with false memory [9,14]. N400 effects are strongly related to semantic/conceptual processing [20], and the effects in the aforementioned studies were of equivalent amplitude for true and false memory. This suggests that they reflected the similar conceptual activation of both studied items and unstudied critical lures during the study phase. These effects could have been identified only in a small minority of long-term DRM experiments because effects on N400 potentials generally reflect conceptual priming over short delays[20], and therefore might be variable in terms of contributions to ERP effects in long-term DRM studies. We thus hypothesized that N400 effects would be especially relevant for false memory in the short-delay DRM paradigm, given that priming effects might be particularly relevant under these testing circumstances (e.g., [21]). Indeed, a recent experiment using fMRI to investigate false memory in a short-delay DRM paradigm identified brain activity consistent with a role for semantic fluency [8]. Notably, the high time resolution of ERPs provides an advantage for the identification of neurocognitive processing relevant to priming, given that relevant brain processing often unfolds with different timecourses within the first several hundred milliseconds of a retrieval cue.

To gain additional interpretive leverage regarding ERP correlates of true and false recognition, we also included a repetition priming manipulation during the test phase. Targets repeated from the study phase as well as unstudied items and related critical lures were repeated after short delays during the test phase. This sort of repetition priming is a common method for isolating neural correlates related to a particular cognitive event of interest (note however that the main behavioral and ERP analyses of false memory effects used only the first presentation of each item, such that neural correlates concerned true and false memory independent from test-phase repetition). In many priming paradigms, for instance, repetition engenders faster and more accurate responding, indicating that the cognitive process supporting the decision was made fluent or less effortful [12,22-26]. Likewise, in fMRI adaptation paradigms, the neural correlates of a cognitive event of interest can be identified because less activity accompanies this event following short-term repetition [26-32].

We therefore reasoned that short-term repetition effects could be used to better map the functional properties of ERP correlates of true and false recognition in the short-term DRM paradigm. For instance, N400 repetition effects tend to reach a maximum value on the second presentation of a word, indicating a plateau in the level of possible conceptual activation [20,33,34]. Furthermore, if late potentials reflect monitoring, then we would expect them to show negative repetition effects only for those conditions for which monitoring occurred (that is, monitoring was needed the first time a response is made to a target or correctly rejected lure, but not the second time). In contrast, monitoring must be relatively minimal or unsuccessful for false alarms, and therefore repetition effects for this condition were hypothesized to be minimal.

## Methods

### Participants

Behavioral and ERP data were collected from 25 Capital Normal University students. All participants were native speakers of Mandarin. Data from four subjects were excluded due to low memory performance (less than 0.80 hits rate to targets). The remaining 21 participants (12 female, 9 male) were 18–25 years old (mean = 23 years), right-handed, and had normal or corrected-to-normal vision. All subjects were paid for their voluntary participation.

### Materials

Visual stimuli included Chinese two-character nouns grouped into 96 lists, each of six conceptual associates. Each list included a theme word and the five most highly related associates, derived from the set of 233 Chinese associate lists reported in [9]. An additional 96 two-character nouns were selected from the Geng et al. (2007) word lists that are conceptually unrelated to the six-item associate lists. Lists were selected such that no word appeared on more than one list. Test words were presented on a computer monitor in white simsun 40-point font against a black background. Words subtended approximate visual angles of 2.2° horizontally and 1.3° vertically from a viewing distance of 1 m. Text in each study list was presented in one of five randomly selected colors (red, yellow, blue, green, or black) to promote the encoding of sensory information [35] and all words in a given list were presented in the same color. This manipulation was included to enhance differences between true and false memory, given that unique color information could exist for true memory but not false memory (see Ref. [35]).

The 96 study lists were categorized according to emotional content, with 64 lists categorized as emotionally neutral, 16 lists as positive, and 16 lists as negative (from [9]). To increase the number of trials available for ERP analysis, words from all lists were pooled. Notably, the prevalence of false alarms to unrelated lures (false memory) did not differ significantly by list type, F(2,40) = 3.3, p > 0.05 (mean = 0.53 for neutral, 0.54 for positive, and 0.58 for negative). This suggests that list emotional category did not have appreciable influences on the reported behavioral and ERP effects.

### Paradigm

**Figure 1:**
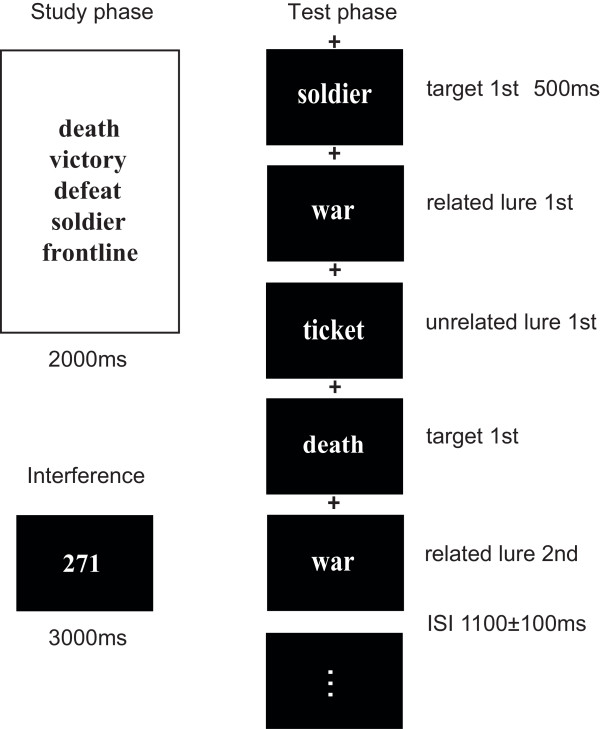
** The short-term DRM paradigm.** Each study phase included an associate list followed by an interference task and then the recognition test. The recognition test included targets as well as related and unrelated unstudied words, all of which were repeated after short delays as part of a repetition priming manipulation.

The short-term DRM task comprised 96 study-test blocks. During each study session, one five-associate word list was presented at the center of the monitor for two seconds (Figure 1). A three-second interference task followed study in which a three-digit number was presented and subjects counted aloud from this number backwards by units of three. The interference task was included to reduce active maintenance (presumably mediated by working memory) of words from study to test.

Memory testing immediately followed the interference task for each study-test block. Two randomly selected studied words (targets) and the unstudied but semantically related theme word (related lure) from each list were presented, along with one semantically unrelated word (unrelated lure), in randomized order (500 ms presentation duration, 1,000–1,200 ms randomized ISI with fixation cross). Each word was presented twice during the test, and two of the words (randomly selected) were presented thrice (10 test trials total). Stimulus presentation order was randomized but with the following constraints: the first three trials always included one studied word (randomly selected), the related lure, and the unrelated lure, presented in randomized order. This ensured that the main true and false memory conditions were presented before any repetition occurred. The remaining stimuli were presented in randomized order following the first three trials. Thrice-presented words were not included in behavioral or ERP analysis, but were included to discourage subjects from anticipating forthcoming words during testing (i.e., it was unclear to subjects how many times a given word would appear). Participants were instructed to indicate whether each word was old or new by pressing one of two buttons with their right or left thumb (assignment of old/new to left/right was counterbalanced across subjects). Speed and accuracy were emphasized. The order of study-test blocks was randomized across subjects.

### ERP methods

Continuous electroencephalogram recordings were collected during the experiment from 62 scalp sites using Ag/AgCl electrodes embedded in an elastic cap at locations conforming to the extended international 10–20 system. These electrodes were referenced to the left mastoid during recording and re-referenced to the average of the right and left mastoid offline. Two additional channels were used for recording electroocculogram. Impedance was less than 5 kΩ, and recordings were made filtered with a bandpass of 0.05–40 Hz and sampled at a rate of 500 Hz. ERPs were computed time-locked to word onset during the memory tests, in 1000-ms epochs starting 100 ms before stimulus onset. Baseline correction was performed using the prestimulus interval. Trials with a voltage, relative to the 100 ms baseline, exceeding ±75 μV at any electrode were excluded from analysis, as were trials with electroocculograph activity indicating eye movements or blinks.

Participants were seated comfortably in an electrically shielded, dimly lit chamber. Participants were instructed to relax their muscles, to blink as little as possible, and to minimize body and eye movement.

ERPs were computed for targets and related lures separately for the two response types (old and new), and separately for the first and second presentations of each word. The mean and range trial counts for ERP averaging are as follows: 1st presentation target hit condition, mean = 133, range = 89–176; 1st presentation related lure false alarm condition, mean = 40, range = 15–71; 1st presentation related lure correct rejection condition, mean = 35, range = 15–54; 2nd presentation target hit condition, mean = 132, range = 78–180; 2nd presentation related lure false alarm condition, mean = 42, range = 23–69; 2nd presentation related lure correct rejection condition, mean = 31, range = 9–55. ERP correlates of true and false memory were computed first independent of repetition (for the first presentation only), and then repetition effects were assessed in a second analysis comparing first to second presentations. ERPs for unrelated lures were not considered (see below). Statistical assessment focused on mean ERP amplitudes averaged in successive 100-ms intervals starting at stimulus onset. Based on the observed characteristics of the ERPs for each condition as well as on prior experiments [9,36,37], statistical assessments were performed on amplitude values averaged over sets of midline electrodes along the anterior-posterior axis (frontal: F3, Fz, F4; fronto-central: Fc3, Fcz, Fc4; central: C3, Cz, C4; centro-parietal: Cp3, Cpz, Cp4; parietal: P3, Pz, P4). Repeated-measures ANOVA (RM-ANOVA) included Greenhouse–Geisser corrections when necessary and Bonferroni-corrected post-hoc pairwise comparison.

## Results

### Behavior

**Figure 2:**
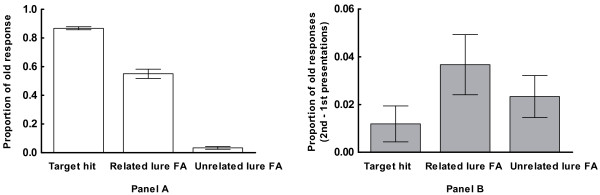
** True memory, false memory, and repetition effects.(a)** Endorsement rates are provided for targets, related lures, and unrelated lures, for the first presentation. **(b)** The difference in endorsement rates for the 2nd minus 1st presentation (repetition effects) is provided for the same conditions. Error bars indicate *SE.*

True and false recognition were examined by comparing the proportion of three categories: “old” responses to targets repeated from the study list (hits) and “old” responses made to related and unrelated lures (false alarms), all seen for the first time during the recognition test. The target hit rate was 0.87 (SE = 0.01), the false alarm rate to related lures was 0.55 (SE = 0.03), and the false alarm rate for unrelated lures was 0.03 (SE = 0.01) (Figure 2A). RM-ANOVA revealed a significant main effect of category [F(2,40) = 448.38, p < 0.001]. Bonferroni-corrected post hoc tests showed that hits to targets were significantly more prevalent than false alarms to either related lures (MD = 0.32,p < 0.001) or unrelated lures (MD = 0.85,p < 0.001), indicating significant veridical recognition of targets. False alarms to related lures were significantly more prevalent than false alarms to unrelated lures (MD = 0.52,p < 0.001), thus demonstrating the usual DRM false memory effect. Notably, false memory responses (false alarms to related lures) were robustly produced by every individual tested, ranging from 26% to 79% (mean = 55%, SE = 3%), indicating that ERP correlates of false memory did not derive from only a subset of individuals.

The effects of repetition during the test on the proportion of true and false recognition were examined for these three categories using RM-ANOVA. There was a category effect collapsing over 1st and 2nd presentations [F(2,40) = 526.46, p < 0.001] (see Figure 2B). A main effect of repetition [F(1,20) = 13.14, p < 0.01] coupled with a non-significant interaction indicated that the prevalence of these three response categories increased with repetition, without significant variability across categories. A main effect of category indicated that the effects described above for the first presentation also held for the second [F(2,40) = 557.72, p < 0.001]. Bonferroni-corrected post hoc tests showed that hits to targets were significantly more prevalent than false alarms to either related lures (MD = 0.29, p < 0.001) or unrelated lures (MD = 0.82, p < 0.001), and false alarms to related lures were significantly more prevalent than false alarms to unrelated lures (MD = 0.53, p < 0.001).

Mean response times (RTs) are presented in Table 1 for correct and incorrect responses to targets, related lures, and unrelated lures, for both the first and second presentations. A significant interaction between condition and repetition [F(5,100) = 5.00, p < 0.05] indicated that the magnitude of priming effects varied across conditions. RTs for second presentations were faster than for first presentations (p values < 0.001) except for false alarms to unrelated lures (p > 0.6). Furthermore, RTs were significantly longer for correct rejections to first presentations of related lures than for first presentations of unrelated lures (MD = 160, p < 0.001), consistent with the greater difficulty of correctly rejecting related lures.

**Table 1:** Reaction times for the three stimulus categories and response types (old/new) in the test phase (mean ± SEM)

	**Target**	**Unrelated lure**	**Related lure**
	**Hit/Miss**	**CR/FA**	**CR/FA**
1st presentation	606 ± 19/756 ± 37	608 ± 19/440 ± 70	769 ± 32/655 ± 30
2nd presentation	519 ± 17/637 ± 26	551 ± 19/484 ± 29	578 ± 23/538 ± 21

CR, correct rejection; FA, false alarm.

### ERP correlates of true and false recognition

**Figure 3:**
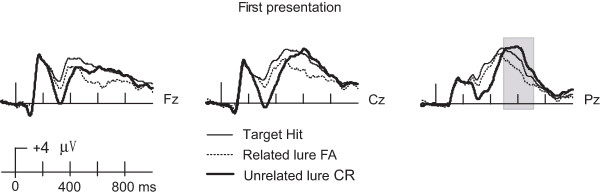
** ERP correlates of first presentation including unrelated lure correct rejections.** ERP waveforms are shown for target hits, related lure false alarms, and unrelated lure correct rejections for three representative midline locations.

To isolate neural correlates of true recognition and of false recognition, we compared ERPs elicited during the first presentation of words during the memory test for three conditions: target hits, related lure false alarms, and related lure correct rejections. Because ERPs were computed for first presentations only, effects described here were independent from the repetition manipulation (effects of repetition on ERPs are described in the next section). ERP correlates of false recognition were identified by comparing false alarms for related lures to correct rejections for related lures, and these ERPs were contrasted to those associated with true recognition (target hits). Using correct rejections of related lures as the “baseline” condition against which to identify effects of true and false recognition is essential because this strategy ensures that all conditions are semantically related. That is, false recognition is defined by the incorrect memory decision for related lures, as compared to correct memory decisions for the same stimulus category. Although the DRM memory illusion is defined behaviorally based on the heightened false alarm rate for related lures relative to unrelated lures, identification of ERP correlates of false memory based on related vs. unrelated lure comparisons is problematic because of the high degree of semantic dissimilarity for unrelated lures (see Figure 3). This dissimilarity produced a large P3 oddball effect for unrelated lure correct rejections for the first presentation. For 600–700 ms, RM-ANOVA for target hits, related lure false alarms and unrelated lure correct rejections at parietal electrode clusters showed that a significant main effect of condition [F(2,40) = 21.72, p < 0.001], whereby the ERPs of unrelated lure correct rejections were more positive than the target hits [MD = 1.72, p < 0.05]. Thus, using unrelated lures as the baseline condition would introduce a stimulus confound that would obscure ERP correlates of false recognition. In contrast, by comparing false alarms to correct rejections for related lures, we were able to isolate the neural processing events associated specifically with the experiences of false recognition for some related lures and of correct recognition (rejection) for other related lures.

**Figure 4:**
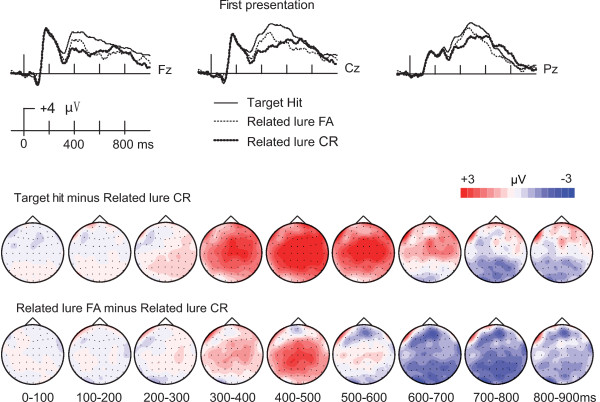
** ERP correlates of true and false recognition.** ERP waveforms are shown for target hits, related lure false alarms, and related lure correct rejections for three representative midline locations. Topographic plots of the ERP difference waveforms are shown on the map of the head, with coloration indicating difference amplitude.

Visual inspection of ERP waveforms (Figure 4) suggested that ERPs for these three conditions diverged in three primary ways: (1) target hits and related lure false alarms both appeared to be more positive than related lure correct rejections from approximately 300–500 ms, whereas (2) only target hits continued to be more positive than related lure correct rejections until about 650 ms. In contrast, (3) related lure false alarms were substantially more negative than correct rejections after about 600 ms, whereas target hits were only slightly more negative than related lure correct rejections after about 700 ms. These ERPs are therefore suggestive of similarities between true and false recognition (early positive ERP effects and later negative ERP effects for both) as well as differences (heightened earlier positivity for true recognition and heightened later negativity for false recognition).

Statistical comparisons for successive 100-ms intervals for the five midline electrode clusters are summarized in Table 2. No reliable differences between the three conditions were identified before 300 ms.

**Table 2:** Statistical comparisons between first presentations of target hits, related lure false alarms, and related lure correct rejections for the five midline electrode clusters in successive 100-ms intervals

	**0–100 ms**	**100–200 ms**	**200–300 ms**	**300–400 ms**	**400–500 ms**	**500–600 ms**	**600–700 ms**	**700–800 ms**	**800–900 ms**
Condition	0.32	0.02	0.33	17.64***	24.27***	15.47***	9.13***	3.61*	1.37
Condition × location	2.09	1.05	1.37	1.35	3.92*	3.10	2.59	4.05*	4.22*

*p < 0.05; **p < 0.01; ***p < 0.001.

ERPs for target hits began to diverge from ERPs for related lure false alarms and correct rejections from 300–400 ms (main effect of condition type, Table 2). During this interval, pairwise comparison indicated target hits were significantly more positive than the other two conditions for all electrode clusters (p values < 0.001), whereas related lure false alarms and correct rejections did not differ reliably at any cluster (p > 0.14).

From 400–500 ms, ERPs for both target hits and related lure false alarms differed from related lure correct rejections, and the nature of these differences varied by location (significant main effect of condition type and significant interaction with cluster, Table 2). For posterior regions (parietal and centro-parietal), target hits and related lure false alarms were both reliably more positive than related lure correct rejections (p values < 0.05), but there were no significant differences between target hits and related lure false alarms (p values > 0.23). More anterior clusters showed different patterns. Fronto-central and central clusters showed a graded effect, with targets hits the most positive and related lure correct rejections least positive (target hits > related lure false alarms > related lure correction rejections(p values < 0.05). In contrast, target hits were significantly more positive than the other two conditions (p values < 0.01), which did not differ significantly (p > 0.09) at the frontal cluster. Thus, positive ERP correlates of true and false recognition were similar at more posterior locations, but these ERP effects were progressively more selective for target hits at the more anterior locations.

The selectivity of positive ERP effects for target hits became more pronounced and was apparent for all locations from 500–700 ms. Significant main effects of condition but no interactions with location were observed for both the 500–600-ms and 600–700-ms intervals (Table 2). Target hits were more positive than both related lure false alarms and related lure correct rejections for 500–600 ms and for all clusters (p values < 0.001). In contrast, there were no reliable differences between related lure false alarms and related lure correct rejections for this interval for any cluster (p = 1). For 600–700 ms, target hits were significantly more positive than related lure false alarms (p < 0.001), but only numerically more positive than related lure correct rejections (p > 0.51). Moreover, related lure correct rejections were marginally more positive than related lure false alarms (p = 0.053) for this interval.

After 700 ms, a different pattern emerged of more negative ERPs for related lure false alarms compared to correct rejections. Interactions between condition and cluster were reliable for the 700–800-ms and 800–900-ms intervals (Table 2). This reflected relatively negative ERPs for related lure false alarms selective for more posterior locations. Related lure false alarms were significantly more negative than correct rejections at parietal and centro-parietal clusters for the 700–800 ms interval (p values < 0.01) and at only the parietal cluster for the 800–900 ms interval (p < 0.05). Target hits were not reliably different from the other two conditions at these clusters for either interval (p values > 0.06). Furthermore, there were no reliable differences between conditions at more anterior clusters (p values > 0.07). Thus, although target hits appeared to show a small trend for more negative amplitudes at posterior locations relative to related lure correct rejections, only related lure false alarms were reliably more negative than correct rejections for these intervals.

### ERP repetition effects during the recognition test

**Figure 5:**
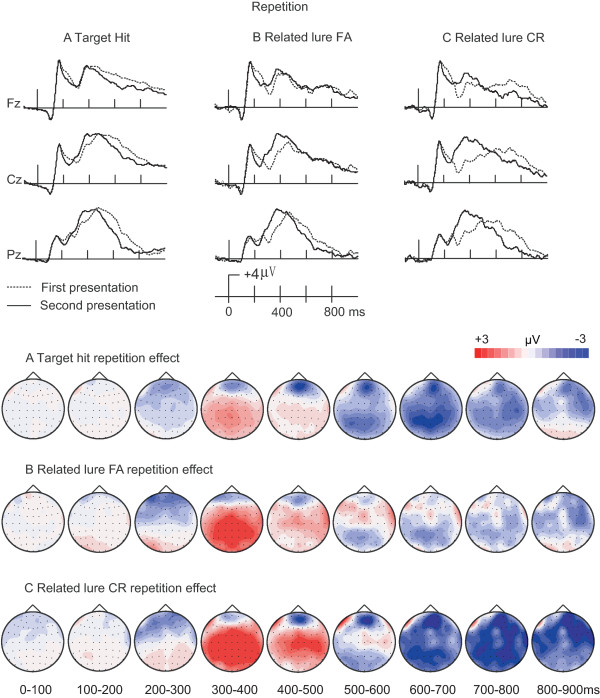
** ERP correlates of repetition priming during recognition testing.** ERP waveforms corresponding to the repetition effect (first versus second presentation) are shown for target hits, related lure false alarms, and related lure correct rejections, for three representative scalp locations. Topographic plots corresponding to the ERP repetition difference are shown for each of the three conditions.

In order to better interpret the functional significance of the aforementioned ERP effects, we examined ERP correlates of short-term repetition during the memory test (see Introduction). We first compared ERPs for the first and second presentation of each word during the recognition test separately for each condition (target hit, related lure false alarm, and related lure correct rejection). As shown in Figure 5, all conditions appeared to exhibit frontally centered negative repetition effects from approximately 200–300 ms, widespread positive repetition effects from approximately 300–500 ms, and varied repetition effects for later latencies. After about 600 ms, negative repetition effects appeared to be present for targets and related lure correct rejections, whereas repetition effects for related lure false alarms appeared to be negligible.

There were no main effects of repetition for any of the three conditions for the 0–200 ms interval (Table 3). Repetition effects for 200–300 ms were negative for all conditions (Table 3). These effects were localized to central, centro-frontal and frontal clusters for target hits (p values < 0.05), and to the frontal cluster for related lure correct rejections and false alarms (p values < 0.05).

**Table 3:** Repetition effects for target hits, related lure false alarms, and related lure correct rejections for the five midline electrode clusters for successive 100 ms intervals

		**0–100 ms**	**100–200 ms**	**200–300 ms**	**300–400 ms**	**400–500 ms**	**500–600 ms**	**600–700 ms**	**700–800 ms**	**800–900 ms**
Target	Repetition	0.002	0.003	6.79*	10.96**	0.18	11.28**	19.37***	8.96**	1.98
Hit	Repetition × location	0.95	0.30	3.84*	12.63***	7.37**	5.55*	3.44	1.34	4.26*
Related lure	Repetition	0.005	0.57	1.42	26.59***	1.98	0.023	0.71	1.15	1.47
FA	Repetition × location	0.28	0.58	9.48**	11.82***	2.06	9.59**	5.91*	2.22	0.71
Related lure	Repetition	0.26	0.003	0.11	18.25***	5.43*	0.32	8.56**	11.85**	6.66*
CR	Repetition × location	5.03*	1.64	12.09***	20.88***	9.96**	3.77*	0.79	0.25	1.26

*p < 0.05; **p < 0.01; ***p < 0.001.

For the 300–400 ms interval, all conditions showed reliable positive repetition effects for most locations (i.e., second > first). Main effects of repetition and repetition-by-location interactions were reliable for all conditions (Table 3). ERPs for the second presentation of target hits were significantly more positive than for the first presentation for all electrode clusters except for the frontal cluster (p values < 0.05; frontal cluster p > 0.44). The same relative positivity for second presentations compared to first was also observed at all clusters for both related lure false alarms and related lure correct rejections (p values < 0.05). Thus, all conditions exhibited widespread positive repetition effects for this interval.

In contrast, positive repetition effects for the 400–500-ms interval were less robust and were relatively selective for related lure correct rejections. Target hits showed a significant interaction between repetition and cluster (Table 3), indicating significant variability in repetition effects across clusters, but the repetition effect was not reliable for any cluster (p values > 0.24). There were no reliable repetition effects for related lure false alarms (Table 3). For related lure correct rejections, a main effect of repetition and significant repetition-by-location interaction (Table 3) reflected reliable positive repetition effects at for all locations (p values < 0.05) except for the parietal (p = 0.07) and frontal (p > 0.59) electrode clusters.

After 500 ms, repetition effects began to appear noticeably negative (i.e., second < first) and varied to some degree across conditions and locations. For the 500–600-ms interval, negative repetition effects were reliable for target hits (Table 3), for all electrode clusters (p values < 0.05). In contrast, negative repetition effects for related lure false alarms (Table 3) were restricted to the parietal cluster (p < 0.05; other clusters’ p values > 0.26). Repetition effects varied reliable across clusters for related lure correct rejections, but there were no reliable repetition effects for any cluster (p values > 0.13).

From 600–800 ms, reliable negative repetition effects were observed for target hits and related lure correct rejections at most locations, but only at restricted locations for related lure false alarms. For the 600–700-ms and 700–800-ms intervals, negative repetition effects were reliable at all electrode clusters for target hits and related lure correct rejections (Table 3; all pairwise p values < 0.01). For related lure false alarms, negative repetition effects were reliable only at the parietal cluster and only for the 600–700-ms interval (p < 0.05; other p values > 0.12).

For the 800–900-ms interval, only related lure correct rejections showed robust negative repetition effects (Table 3) that were reliable for all electrode clusters (p values < 0.05). A significant repetition-by-location interaction for target hits reflected reliable variability in repetition effects across locations, but repetition effects were unreliable at all individual clusters (p values > 0.07). There were no reliable effects for related lure false alarms (Table 3).

## Discussion

In a short-term DRM paradigm participants exhibited typical false-memory effects by endorsing more related lures as studied than unrelated lures. Furthermore, a standard repetition effect was identified for all stimulus categories during the test, with faster responses for the second presentation than the first presentation. ERPs collected during memory testing illuminated the neural basis of short-term false memory effects, which we now summarize via comparison to ERP correlates of memory-related neurocognitive processing, emphasizing similarities and differences with false long-term memory.

As hypothesized, clear N400 old/new effects were identified from 300–500 ms for target hits (true recognition) and for related lure false alarms (false recognition), both relative to related lure correct rejections, but with some variation across these memory outcomes. Whereas the positive N400 effect was robust for true recognition from 300–400 ms, the positive N400 trend for false recognition did not reach statistical significance for this interval. N400 effects from 400–500 ms were robustly reliable for both memory outcomes, but with minor distributional differences. Whereas both true and false recognition were associated with reliable N400 effects at posterior electrode locations, only true recognition produced reliable effects at more anterior locations. Based on previous findings regarding the N400 [20], we interpret the current N400 effects as indicative of contributions from semantic/conceptual activation to recognition judgments, as identified in only a small minority of long-term DRM experiments [9,14]. This distinction provides preliminary evidence that short-term and long-term DRM false memory effects rely on distinct neurocognitive processing, with a greater emphasis on semantic/conceptual activation and fluency (priming) for short-term false memory. Although long-term false memory effects are unlikely due to semantic/conceptual priming, both short- and long-term false memory effects could involve an influence from semantic/conceptual activation for unstudied items during study. That is, in both cases, subjects may produce (covertly activate) the unstudied critical/related lure while reading the study list, and this activation could promote subsequent false memory. In long-term tests, false memory might be more likely to derive from monitoring failure, whereby covert activation from study is confused with real study experiences due to source memory error. In contrast, false memory in short-term tests might be more likely to arise from short-lived semantic/conceptual priming due to the covert activation. Testing this possibility will require neurocognitive measures of both short-term and long-term false memory in similar testing circumstances and in the same individuals.

We also identified positive old/new effects from 500–700 ms that were similar to LPC effects identified in previous studies in relation to true recognition (see Introduction). These effects likely reflected the retrieval of specific sensory details from the study phase (including perhaps word coloration information, which was uniquely present for studied words). False memory also likely involves the retrieval of episodic details, but not the sensory details that are available only for studied targets. LPC potentials are associated with the experience of recollection, which occurs when details from study are vividly “relived” during retrieval [38]. Thus, sensory details were potentially re-activated only for true recognition as reflected by these potentials (see also the sensory reactivation hypothesis of [39]).

ERP effects after 700 ms were related to monitoring process rather than to activation. A negative ERP effect was identified for false recognition, but not for true recognition. False recognition represents a failure of retrieval monitoring, relative to the successful retrieval monitoring associated with correctly rejecting a related lure. Thus, the ERPs reflect this monitoring failure by demonstrating lower amplitudes of the late positive ERPs normally associated with retrieval monitoring (see Introduction) for the false recognition condition. Long-term DRM paradigms have variably reported late ERP effects (after 800 ms), including effects that are either more positive for false compared to true memory, or more negative [9,11,40]. Because our findings concern false short-term memory, it is unclear how the late positive effect we identified relates to these previous findings. One possibility is that DRM parameters, which vary widely across ERP experiments, differ in their promotion of monitoring processes reflected by late-onset ERPs. Our findings suggest that short-term false memory stems from semantic priming (as indicated by N400 effects) occurring along with failure to respond based on the differences in detail retrieval that is greater for true than for false memory (and reflected by LPC-like ERPs) and less effortful retrieval and/or retrieval monitoring (reflected by post-800 ms ERPs). Future research should attempt to identify how different DRM parameters systematically modulate the relative emphasis on these different processes, such that the mechanisms for false memory can be better understood.

**Figure 6:**
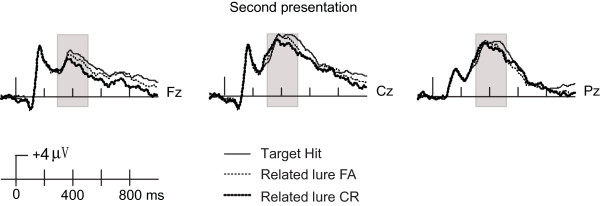
** Saturation of N400 correlates of activation.** ERPs for the second presentations of target hits, related lure false alarms, and related lure correct rejections are shown together at three representative locations in order to emphasize the saturation of N400 correlates of activation by the second presentation.

These functional interpretations of ERP effects identified during the recognition test were further supported by results from the test-phase repetition manipulation, at least for N400 effects and for late effects related to monitoring (note that the aforementioned ERP correlates of true and false recognition were independent from repetition as they concerned only the first presentation of words in each condition). All conditions exhibited positive N400 repetition effects during test. Notably, however, the overall magnitude of the N400 was matched across the true and false recognition conditions for the second presentation during the test [F(1,20) = 0.016 for 300–400 ms; F(1,20) = 2.29 for 400–500 ms; p values > 0.15;] (Figure 6). Thus, the N400 showed a “saturation” effect, presumably reflecting the maximal semantic/conceptual activation possible upon the second test-phase presentation (see Introduction). Notably, because N400 effects varied somewhat for true and false recognition upon the first presentation, the repetition effects were somewhat variable for these conditions, such that similar amplitudes were achieved upon second presentation. This saturation of semantic activation is strongly consistent with the interpretation that N400 effects reflected priming of study-phase semantic activation relevant to true and false memory decisions.

Repetition effects on ERPs also supported the interpretation of late effects as ERP correlates of retrieval monitoring. If late effects were indicative of retrieval monitoring, then we would expect negative repetition effects only for the conditions that initially involved retrieval monitoring on the first presentation. That is, if retrieval monitoring occurred during the first presentation, then it would not be required on the second presentation (which occurred after a brief delay from the first presentation), thus leading to a negative repetition effect (i.e., less of the late positive ERPs associated with monitoring). Indeed, negative repetition effects after 500 ms were noted for both target hits and related lure correct rejections, the two conditions involving retrieval monitoring, but not for related lure false alarms, the condition involving a failure of retrieval monitoring. Further, both ERPs of target hits and related lure correct rejections were matched with the related lure false alarms at the second presentation after 600 ms (p values > 0.5). This solidifies the relationship between late ERP effects and retrieval monitoring, and furthermore indicates that the late negative ERP correlate of false recognition (Figure 4) was indeed indicative of monitoring failure for this condition, as hypothesized above. It should be noted that the prefrontal cortical regions likely involved in retrieval monitoring have been shown to exhibit negative repetition priming effects [41], thus supporting our interpretation of negative repetition effects as indicating less retrieval monitoring. Although we used repetition priming as a way of gaining interpretive leverage on ERP correlates of true and false memory, other methods could provide similar information in future studies, such as measures of self-reported illusory recollection or confidence[6].

## Conclusions

To summarize, we identified neurocognitive processing related to false memory in a short-term variant of the DRM paradigm. Our novel repetition priming manipulation during the test phase allowed us to make relatively strong functional interpretations of the resultant ERP effects. In doing so, we identified N400 effects indicative of semantic priming related to both true and false memory. In addition, we showed that late ERP correlates of retrieval monitoring are produced only under conditions when monitoring is required and is effective. Our results converge with those from a recent fMRI experiment that investigated neural correlates of true and false memory in a short-delay DRM paradigm [8]. In that experiment, prefrontal cortical activity provided evidence for the role of semantic activation/priming in short-term false memory, in that relative decreases in activity for false memory suggested a failure to inhibit semantic fluency for falsely recognized related lures using frontally mediated control processing. Our results are consistent with these interpretations, with the N400 ERP correlates of false memory reported here reflecting the semantic fluency that is presumably inhibited by the later-occurring ERP effects in true versus false memory.

## Competing interests

The authors declare that they have no competing interests.

## Authors’ contributions

Both HC and CYG took part in planning and designing the experiment. JLV and HC completed the data collection and analyses and drafted the manuscript. CYG assisted in preparing the manuscript. All authors read and approved the final manuscript.
